# Flame Retardation of Natural Rubber: Strategy and Recent Progress

**DOI:** 10.3390/polym12020429

**Published:** 2020-02-12

**Authors:** Le Wan, Cong Deng, Ze-Yong Zhao, Hong Chen, Yu-Zhong Wang

**Affiliations:** Analytical & Testing Center, The Collaborative Innovation Center for Eco-Friendly and Fire-Safety Polymeric Materials, National Engineering Laboratory of Eco-Friendly Polymeric Materials (Sichuan), State Key Laboratory of Polymer Materials Engineering, Sichuan University, Chengdu 610064, China; 2017229050008@stu.scu.edu.cn (L.W.); zeyongzhao@scu.edu.cn (Z.-Y.Z.); 2016322030043@stu.scu.edu.cn (H.C.); yzwang@scu.edu.cn (Y.-Z.W.)

**Keywords:** natural rubber, halogen-containing material, halogen-free flame retardant, inorganic compound, nanoparticle, burning

## Abstract

Natural rubber (NR) as a kind of commercial polymer or engineering elastomer is widely used in tires, dampers, suspension elements, etc., because of its unique overall performance. For some NR products, their work environment is extremely harsh, facing a serious fire safety challenge. Accordingly, it is important and necessary to endow NR with flame retardancy via different strategies. Until now, different methods have been used to improve the flame retardancy of NR, mainly including intrinsic flame retardation through the incorporation of some flame-retarding units into polymer chains and additive-type flame retardation via adding some halogen or halogen-free flame retardants into NR matrix. For them, the synergistic flame-retarding action is usually applied to simultaneously enhance flame retardancy and mechanical properties, in which some synergistic flame retardants such as organo-montmorillonite (OMMT), carbon materials, halloysite nanotube (HNT), etc., are utilized to achieve the above-mentioned aim. The used flame-retarding units in polymer chains for intrinsic flame retardation mainly include phosphorus-containing small molecules, an unsaturated chemical bonds-containing structure, a cross-linking structure, etc.; flame retardants in additive-type flame retardation contain organic and inorganic flame retardants, such as magnesium hydroxide, aluminum hydroxide, ammonium polyphosphate, and so on. Concerning the flame retardation of NR, great progress has been made in the past work. To achieve the comprehensive understanding for the strategy and recent progress in the flame retardation of NR, we thoroughly analyze and discuss the past and current flame-retardant strategies and the obtained progress in the flame-retarding NR field in this review, and a brief prospect for the flame retardation of NR is also presented.

## 1. Introduction

**Scheme 1:**
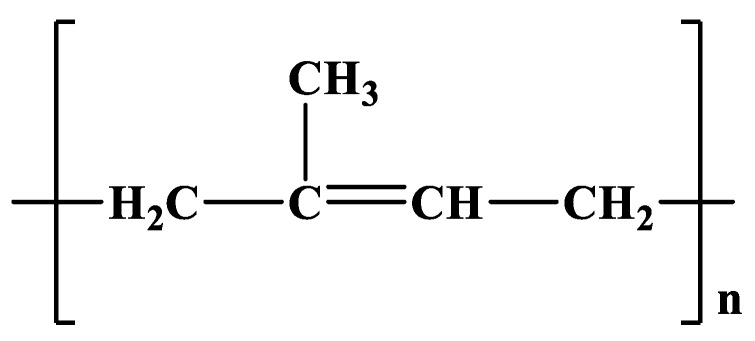
Structure of *cis*-1,4-polyisoprene.

**Figure 1:**
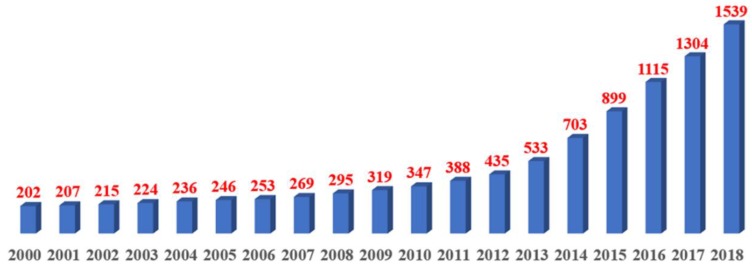
Open documents concerning flame-retarding natural rubber (NR) from 2000 to 2018. (All data were obtained from Web of Science and CNKI; the key words are “flame retard” and “natural rubber”.).

Natural rubber (NR) is a kind of natural polymer that can be obtained from rubber trees, in which 91–94% of component is hydrocarbon (*cis*-1,4-polyisoprene, Scheme 1), and the rest mainly consists of non-rubber substances such as protein, fatty acid, ash, sugar, etc. [1]. NR possesses many excellent properties such as low dynamic response, low hysteresis, high insulation, high durability, and so on [2]. Therefore, it is widely used in daily life, medical and health industry, transportation, agriculture, meteorological measurement, etc. Despite a number of advantages for NR, its inherently high flammability limits its wider usage in some important fields such as conveyor belts for coal mine, power cables, tire treads, etc. [3]. According to the past test [4,5,6], the limiting oxygen index (LOI) of pure NR is only about 18.0%. Therefore, NR is flammable. Moreover, it will emit a high yield of dense smoke and toxic gases once being ignited, which seriously decreases the possibility to escape and thus bring about a great threat to the life and property of people. So far, much attention is paid to the flame retardation of NR. Statistical results show that the total number of open articles and patents for flame-retarding NR gradually increased in the past 20 years. As shown in Figure 1, the total piece of open articles and patents was about 202 in 2000. By the end of 2018, it greatly increased to about 1539. Clearly, in the flame-retarding NR field, much progress has been made in past research.

At present, the most common method for the flame retardation of NR is to incorporate additive-type flame retardants into the matrix, and the category for additive-type flame retardants is very plentiful, including halogenated additives, metal hydroxide, phosphorus-containing flame retardants, intumescent flame retardants, nano flame retardants, etc. Actually, different flame retardants have their own advantages and disadvantages. For instance, inorganic flame retardants such as magnesium hydroxide and aluminum hydroxide are “green” environmentally friendly flame retardants. However, their flame-retarding efficiency is not high. Although some halogenated flame retardants are highly efficient, they have a serious ecological accumulation feature and produce dangerous toxic substances during burning, having a great influence on the environment and human health. For the intrinsic flame retardation of NR, the preparation process of flame-retarding NR is very complicated to be industrialized. Nano flame retardants have a unique advantage in maintaining the mechanical properties of NR, but its flame-retarding efficiency is not ideal. In summary, it is an important issue to develop effective flame-retardant strategies or halogen-free highly efficient flame retardants that may simultaneously endow NR with flame retardancy and excellent other properties. In the following part, the past and current strategy and progress in the flame retardance of NR will be introduced in detail.

## 2. Thermal Decomposition and Burning Behavior of NR

### 2.1. Thermal Decomposition of NR

**Scheme 2:**
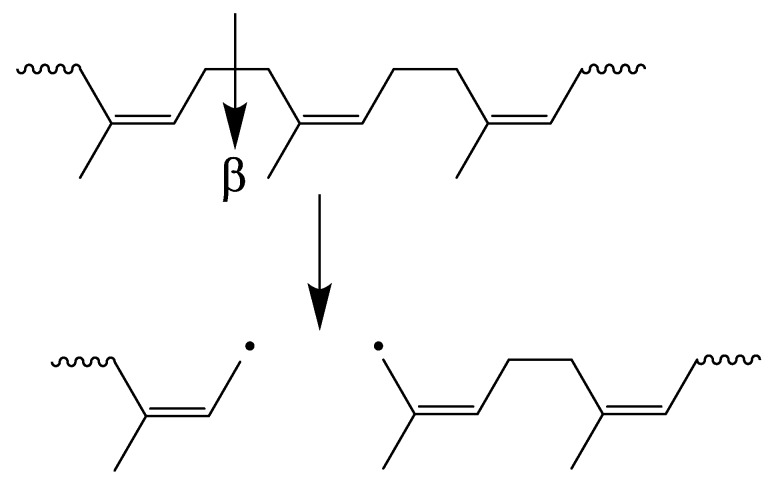
The β-scission of *cis*-1,4-polyisoprene [12].

**Figure 2:**
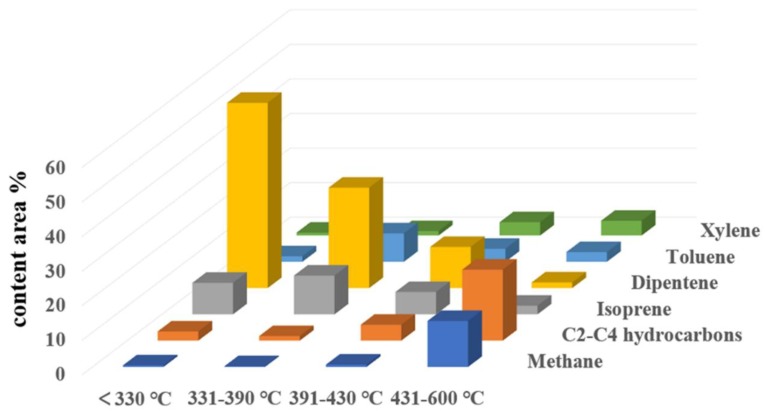
The yield of pyrolysis products of *cis*-1,4-polyisoprene (content area %) at different temperature ranges [12].

**Scheme 3:**
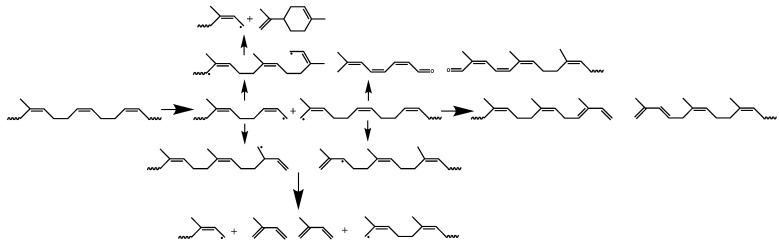
Thermally decomposing process of NR [14].

The thermal decomposition behavior of NR was first discovered in 1860 [7]. The research result shows that several factors might have an important influence on the thermal decomposition of NR, in which the temperature is the most important one. During heating, random chain scission of NR is dominant. By increasing the temperature, the bonds of NR chains would break randomly. Generally, the initial break occurs at the “weak link” because of the existence of some defects. Colin et al. [8] studied the long-term thermal oxidation decomposition of the un-stabilized and un-vulcanized polyisoprene rubber in the temperature range from 40 to 140 °C, and the result showed that the macromolecular chains broke rapidly because hydrogen atoms were replaced by the initially produced free radicals, and then a large amount of oxygen molecules attacked main chains of NR, accelerating the thermal decomposition of NR. Midgely et al. [9] further studied the thermal decomposition of NR and considered that the produced dominant matter was dipentene during heating, accompanied by the appearance of isoprene monomer. For the formation of dipentene, two possible reasons are as follows: one is due to the Diels–Alder addition reaction between two isoprene molecules; the other one is from the back-biting reaction of the pyrolysis product of polyisoprene [10]. Moreover, the yield of isoprene or dipentene is different with changing the heating temperature and other conditions. The higher the temperature is, the higher the yield of isoprene is [11]. Chen et al. [12] also investigated the effect of temperature on the yield of main decomposition products and analyzed the thermally decomposing mechanism of NR. They thought that the β-scission took place firstly because of the low bond-dissociation energy, and then led to the formation of two allylic radicals, as shown in Scheme 2. For the formed allylic radicals, they are prone to forming six-membered rings, leading to a higher yield of dipentene. Figure 2 shows the information on the decomposition products of NR in different temperature ranges. With increasing the temperature above 330 °C, some products became more, such as CH_4_, C2 hydrocarbons, C3 hydrocarbons, C4 hydrocarbons, etc., but some products became less, such as toluene, dipentene, trimeric isoprene, etc. Reshetnikov et al. [13] also studied the thermal decomposition products of synthetic isoprene rubber (SIR), and the result shows a similar trend to that reported by Chen et al. [12]. In addition, Cataldo et al. [14] did deep research via electronic spectroscopy and FT-IR, and they thought that chain scission occurred at the carbon–carbon single bond and the formed allylic radicals might cause two different reaction pathways. As shown in Scheme 3, the first one is the pyrolysis accompanied by the formation of isoprene and dipentene at high temperatures; the second one is that adventitious agents stopped or terminated the allylic radicals at mild conditions. Obviously, the reaction at the first step is consistent with that proposed by Midgely et al. [9].

### 2.2. The Burning Behavior of NR

**Scheme 4:**
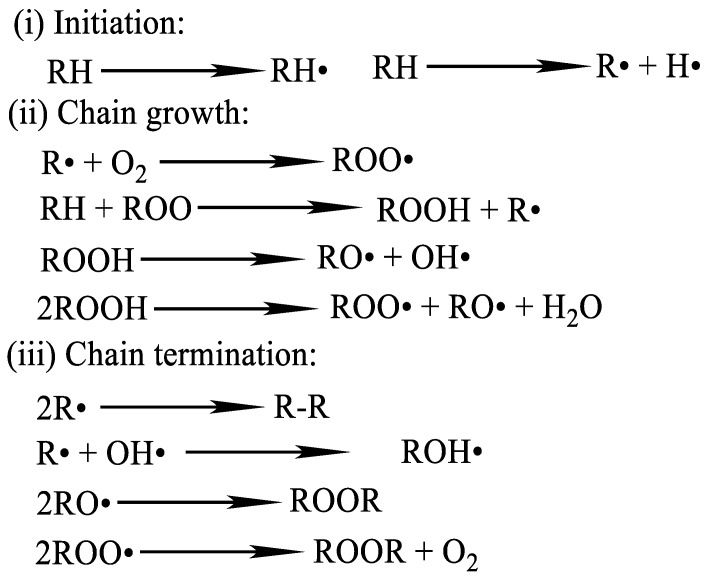
Free radical chain reaction of NR [15].

The molecular chain of NR is made up of carbon and hydrogen, like that of polyethylene (PE) or polypropylene (PP). The past research illustrates that the material possessing this kind of structure is highly flammable, and it burns rapidly once being ignited and the flame spreads quickly, accompanied by the release of a large amount of toxic smoke and gases that may cause great harm to the human beings. The burning behavior of NR is very complicated. In the viewpoint of chemical reaction, the combustion process belongs to a free radical chain reaction, including three stages: (i) chain initiation; (ii) chain growth; (iii) chain termination. When the substrate is heated by an external heat source, some chemical bonds begin to break once the heat source at the surface of the substrate accumulates to a certain high temperature, and the oxidation reaction of molecular chains is an automatic catalytic process under the oxygen-containing atmosphere. Therefore, the reaction for the break of macromolecular chains is prone to undergoing once the burning is triggered. The free radical chain reaction is shown in Scheme 4, as follows [15].

In detail, polyisoprene mostly decomposes via random chain scission during heating, leading to the generation of combustible volatile with low molecular weight, and the volatile may diffuse in the air and result in the formation of a combustible mixture. Once the auto-ignition temperature is reached, the gaseous mixture will be ignited and the burning will also be triggered. Moreover, the released heat may act as a new heat source, and the produced heat is continuously transferred to the material underneath from the contact surface that continues releasing the heat source, then the thermal decomposition and ignition processes are repeated. Finally, the burning process may continue because the fuel can be compensated via the continuous generation of combustible gases during burning. A loop can be formed in this case, and the burning becomes uncontrollable.

## 3. Laboratory Fire Test

To characterize the flame retardancy of materials, a series of test methods and corresponding standards have been established by different organizations or institutions, and the most typical and important test methods for NR includes UL 94 vertical burning standard test, limiting oxygen index test, and cone calorimeter test. 

### 3.1. UL 94 Vertical Flammability Standard Test

**Figure 3:**
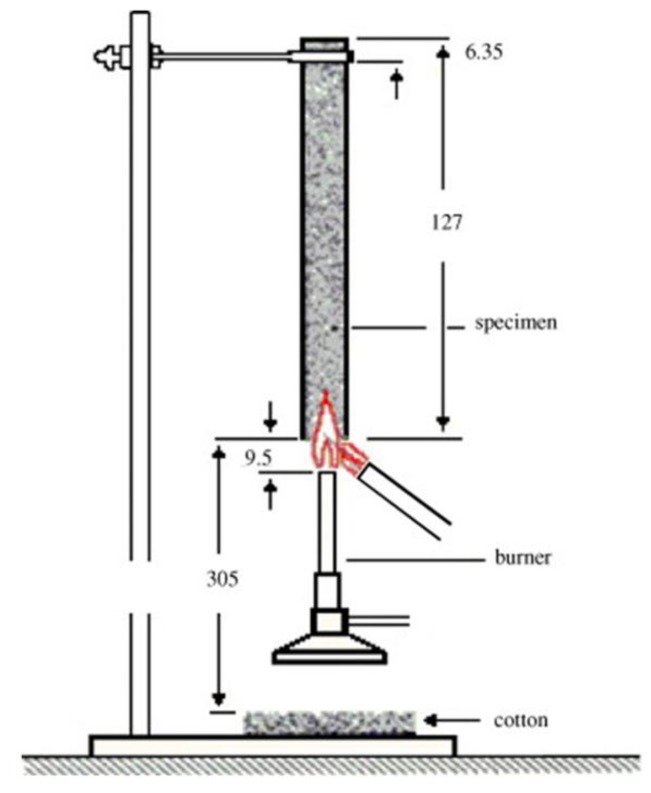
The scheme for the UL 94 vertical burning test [16].

According to the placement state of the sample during testing, the UL94 flammability test can be divided into the horizontal burning test and vertical burning test. For the former, it has a stricter requirement than the latter. For NR products, the vertical burning test is generally applied to measure their flame retardancy, as shown in Figure 3. In the UL 94 vertical burning test, the burning ratings are classified as V-0, V-1, V-2, and no rating, and the detailed criteria are listed in Table 1.

### 3.2. Limiting Oxygen Index

**Figure 4:**
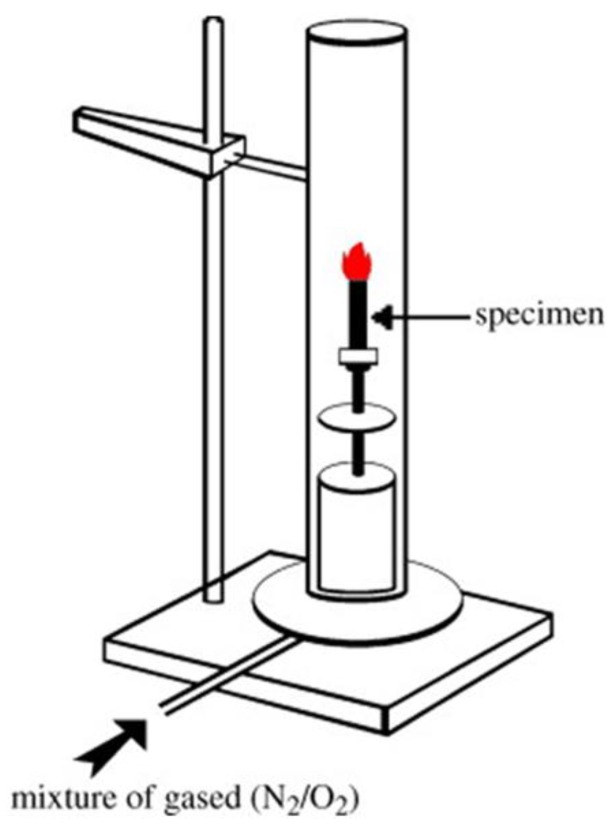
The scheme for the limiting oxygen index (LOI) test [16].

The value of the LOI is defined as the minimum oxygen concentration in the oxygen/nitrogen mixture that either maintains the combustion of material for 3 min or consumes a length of 5 cm of the sample. In the LOI test, the sample is placed in a vertical position (Figure 4). Generally, the higher the LOI is, the better the flame retardancy is. For the material with the LOI value below 21%, it belongs to the “combustible” material; conversely, it is defined as the “self-extinguishing” material. The calculation formula of LOI is:

$$\mathrm{LOI}\left(\%\right)=\frac{\left[O_{2}\right]}{\left[O_{2}\right]+\left[N_{2}\right]}\times 100\%$$

[O_2_] is the flow rate of O_2_, L·min^−1^; [N_2_] is the flow rate of N_2_, L·min^−1^.

### 3.3. Cone Calorimeter

The principle of the cone calorimeter experiment is based on the measurement of the decreasing oxygen concentration in the combustible gases of a sample subjected to a given heat flux (from 10 to 100 KW/m^2^). According to the standard test, the material releases 13.1 KJ of heat when 1 g of oxygen is consumed during the combustion process, and this value is constant and independent of the category of material [17]. At present, the cone calorimetry test is the most effective method to simulate actual fire conditions using the medium-sized sample studying the burning behavior of materials in laboratory. According to the heat and smoke parameters provided by the cone colorimeter test, the flammability of materials can be evaluated quantitatively, mainly including the following results.

(1) Time to ignition (TTI, s): under the same irradiation power and sample thickness, the longer TTI means it is harder to ignite the material. For flame-retarding polymers, they often decompose in advance due to the addition of flame retardant, thereby shortening the TTI. Therefore, the shorter TTI does not mean that the flame retardancy of materials becomes worse.
(2) Heat release rate (HRR, KW/m^2^): it is defined as the heat release per unit time and unit surface area during the cone calorimetry test. Particularly, the peak value of HRR (PHRR) or its maximum (HRRmax) is used to evaluate the fire performance of materials.
(3) Total heat release (THR, kJ/m^2^): the total calorific value released per unit area after the combustion process for materials, which can be calculated according to the integration of the HRR vs. time.
(4) Fire growth rate (FGR, KW/(s·m^2^)): the FGR is expressed as FGR = PHRR/t_PHRR_ [18], t_PHRR_ is the time to reach the PHRR. The lower the FGR value is, the better the fire performance of the material is.
(5) Mass loss rate (MLR, g/s) is the mass loss of material per unit time during burning.
(6) Smoke product rate (SPR, m^2^/s) and total smoke production (TSP, m^2^) reflect the combustion degree of materials.

## 4. The Flame-Retarding Action Mode

### 4.1. Physical Action 

(1) Cooling. Some flame retardants would absorb heat when they decompose, and the endothermic decomposition may consume the released heat from the combustion of material, then the burning material could be cooled to some extent. Generally, most inorganic hydrated compounds like aluminum hydroxide (ATH) and magnesium hydroxide (MH) may play a role via this mode. Table 2 shows the typical endotherm compounds which may absorb the produced heat via decomposing during burning and then achieve the flame retardance of materials.

(2) Barrier action via the formed protective layer. The decomposition products of some flame retardants would shield the surface of the substrate and form a protective layer that may act as a barrier to resist oxygen and the produced heat. As a result, the burning process is difficult to sustain.

(3) Fuel dilution. Some flame retardants may release the water vapor, CO_2_, or other inert gases, and then result in the decrease of the concentration of free radicals and combustible gases in the burning zone. When the concentration of combustible gas decreases to some extent, the burning may be cut due to the lack of fuel.

### 4.2. Chemical Action

(1) Gas phase reaction: the gas phase reaction mechanism is generally regarded as the interruption to the chain reaction during burning. The flame retardant that plays the flame-retarding action via the gas phase reaction action may capture free radicals to make the concentration of free radicals decrease to a value lower than the combustion threshold and then prevent or delay burning, in which halogen-containing flame retardants are the most representative. During burning, halogen-containing flame retardants released the hydrogen halide, which may react with the free radicals which are formed during burning and then inhibit the combustion of substrates. The following equation (Equation (1)) shows the action mode of hydrogen halide.

(1) $$H∙+\;\mathrm{HX}\;⇌{\;H}_{2}\;+\;X$$

(2) Condensed phase reaction: the flame retardant that plays the flame-retarding role via the condensed phase reaction may promote the formation of a carbonized or vitreous layer by crosslinking, aromatizing, catalytically dehydrating for polymers, or reacting with the substrate/the decomposition products of the substrate. In this flame-retardant mode, intumescent flame retardants may form an intumescent char layer by some chemical reactions during burning, and generally, the formed char layer may promote the barrier action and improve the flame retardancy of the substrate. In addition, some flame retardants can accelerate the rupture of polymer chains, and a large number of droplets are produced under this condition, then a large amount of heat may be taken away when these droplets move away from the burning zone. The melt-dripping flame-retardant mode is also a kind of typical flame-retarding route that is caused by the condensed phase reaction.

## 5. Flame Retardance of NR

According to the past report, there are two typical approaches that can reduce the flammability of NR, including intrinsic flame retardation through the incorporation of some flame-retardant unit into polymer chains and additive-type flame retardation via adding some halogen or halogen-free flame retardants into the NR matrix. In addition, synergistic flame retardation in which some synergistic flame retardants such as OMMT, carbon materials, HNT, etc., can be utilized to promote the flame-retarding efficiency of intrinsic or additive-type flame retardation. Generally speaking, different flame-retardant modes have their own advantages. For intrinsic flame retardation, the prepared flame-retarding polymer materials have excellent overall performance, namely, flame retardancy, mechanical properties, durability, etc., which maintain at a high level. However, the preparation for intrinsic flame-retarding polymers is limited due to the lack of a reactive flame-retardant monomer, complex reaction steps, and an inflexible synthesis method. For additive-type flame retardation, the advantage is as follows. The fabrication process for flame-retarding systems is simple, the flame-retarding efficiency can be adjusted easily, the flame-retardant mode is plentiful, etc. However, a major shortcoming is that the mechanical properties of polymers are always seriously deteriorated after the incorporation of a flame-retardant additive. As for synergistic flame retardation, there is no apparent drawback, moreover, the flame-retarding efficiency of intrinsic and additive-type flame-retarding systems may be remarkably promoted in the presence of a small amount of synergistic flame retardants. Actually, both chemical and physical actions always exist simultaneously in many cases. In the following part, the main flame-retardant methods and flame retardants will be overviewed in detail.

### 5.1. Intrinsic Flame Retardation

In recent years, much attention has been paid to the intrinsic flame retardation due to the excellent overall performance of intrinsic flame-retarding polymers, which involves totally new flame-retarding polymers and the modified polymers. For the latter, they are usually prepared by copolymerizing existing polymers with flame-retardant units or using the flame-retardant units as pendent groups along the chain [21]. In terms of NR, researchers prefer to incorporate some flame-retardant units in the backbone because of the high reactivity of C=C in the polyisoprene. In this case, the flame-retardant units may become part of polymer chains via polymerization. In addition, the graft of a flame-retardant unit as side chains of the backbone via the covalent bond is very useful to improve the flame retardancy of NR [2]. Compared with the equivalent additive flame retardant, the flame-retardant units covalently bonded to the molecular chain may distribute uniformly in the polymer matrix, and the flame-retarding effectiveness is usually higher than traditional additive flame retardants. 

**Scheme 5:**
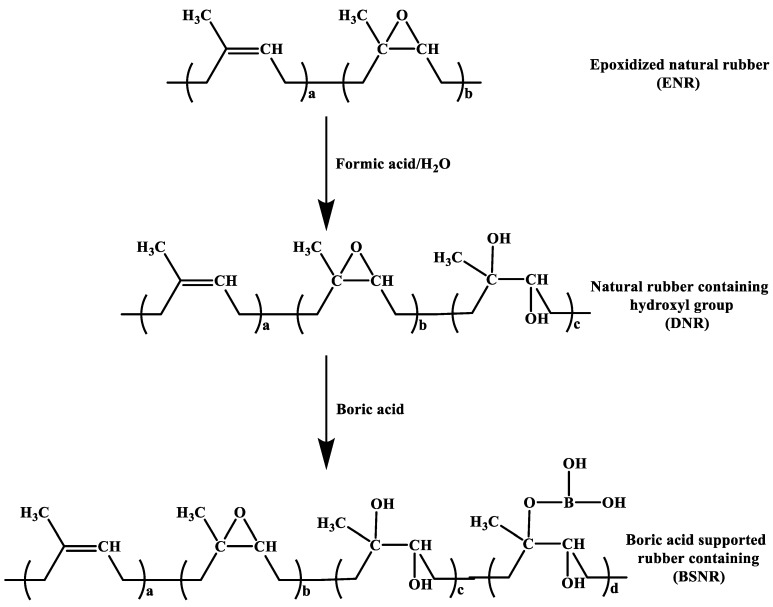
Synthesis procedure for the boron-supported NR (BSNR) [22].

The boron element is efficient to improve flame retardancy via the formation of a hard glassy condensed phase. Intharapat et al. synthesized a new flame-retarding NR through a simple chain reaction, as shown in Scheme 5, in which boric acid was introduced in the NR containing a precursor of reactive hydroxyl groups. Here, the precursor was prepared by the ring-opening reaction of epoxidized natural rubber [22]. For the boron-supported NR (BSNR), its flame retardancy is apparently superior to that of NR. In the cone colorimeter test, the values in mass loss rate (MLR), total heat release (THR), effective heat of combustion (EHC), and the maximum heat release rate (HRRmax) significantly reduced. The research result confirmed that the improvement in flame retardancy after the incorporation of boric acid was due to the promotion of dehydration and the formed protective glassy coating. Actually, boron compounds are lowly toxic, low cost, and present a weak environmental impact [23]. Therefore, the application of boron compounds in the flame-retardant field may become a trend.

**Scheme 6:**
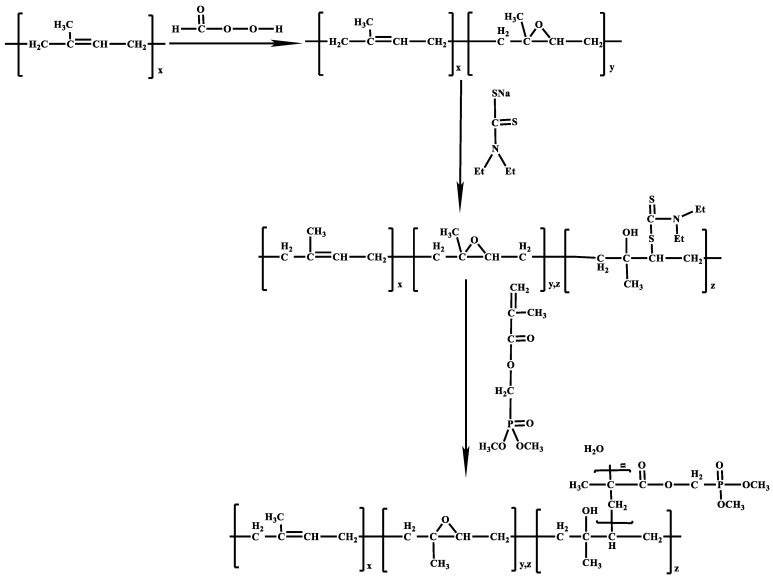
Schematic outline for the synthesis of NR-*g*-PDMMMP [21].

For NR, graft copolymerization via latexes medium is an effective method to endow it with flame retardancy. In Intharapat et al.’s report [21], NR grafted with poly(dimethyl-(methacryloyloxymethyl)phosphonate) (NR-*g*-PDMMMP) was prepared in latex medium via photopolymerization (Scheme 6), and they found that the increase in graft rate (GR) made the decomposing temperature of NR-*g*-PDMMMP rise. When the GR was 95%, the burning rate was decreased by nearly 70%, and the LOI was also improved significantly. Mechanism analysis illustrated that the improvement in flame retardancy was mainly attributed to the formation of char residue which consists of phosphorus compounds. During burning, the phosphorous-containing char residue acted as the thermal insulation and a barrier of oxygen to transfer to the burning materials. Kokklin et al. [24] synthesized a phosphorus-containing monomer named methacryloyloxyethyl phenyl benzenephosphonate (MPBP) and grafted the MPBP onto NR particles via emulsion polymerization. The result showed that the LOI value of NR increased to 20.5% when the monomer content of MPBP was as high as 20.0 wt.% (the content of organic phosphorus was only 1.8 wt.%). 

In intrinsic flame retardation, even though a large amount of flame-retardant monomer is introduced in the NR by copolymerization or graft, the content of the effective element is usually low. When further increasing the content of flame-retardant monomer, other properties of the polymer may be deteriorated or it is too difficult to prepare the macromolecular polymers. Therefore, the intrinsic flame retardation is not dominant in flame-retarding NR even though there are some remarkable advantages.

### 5.2. Additive-Type Flame Retardation

Additive-type flame retardants are generally incorporated into NR by the physical blending method [25]. The most remarkable advantage for the additive flame-retardant method is that the flame retardancy of polymers can be endowed in a simple way. Generally, additive flame retardants are incorporated into the NR by a two-roll mixing miller or an extruder. The processing temperature of NR is usually low, and even it can be processed at room temperature. In addition, the vulcanization temperature is about 140–160 °C. Therefore, the flame retardants will not decompose during processing due to the low processing temperature. So far, according to the practical requirement, traditional halogen-containing flame retardants, inorganic flame retardants, or organic flame retardants may be used to fabricate additive-type flame-retarding NRs. In the following part, the application of different additive flame retardants in NR will be introduced in detail.

#### 5.2.1. Halogen-Containing Flame Retardants

Halogen-containing flame retardants have been widely used for decades because of their high flame-retarding efficiency. Researchers have carried out systematic and in-depth research on its detailed flame-retardant mechanism. The chemical reaction for polymers during burning belongs to a free radical chain reaction, and the continuous growth of free radicals is an important reason for maintaining the burning process for polymers. Halogen-containing flame retardants act by interfering with the radical reaction of chains taking place in the gas phase [26]. In the presence of halogen-containing flame retardants, free radicals can be captured during burning and then the burning behavior of polymers can be prevented. 

In fabricating halogen-containing flame-retarding polymers, the most commonly used flame retardant is chlorinated paraffin (CP). However, the addition of CP alone had little effect on the flame retardancy of NR. Actually, when there is only CP, the CP may make the viscosity of NR increase and then promote the anti-dripping performance during burning, which may lead to the accumulation of heat in the burning zone and further result in the worse flame retardancy of NR. Therefore, little effect on the flame retardancy of NR mentioned above does not mean that the CP is not efficient in fabricating flame-retarding NR. In many cases, the antimony trioxide is used as a synergistic agent. When 25 phr of antimony oxide and 50 phr of CP (70% Cl) were added in NR, NR was rendered non-burning [27]. If 20 phr of zinc hydroxystannate (ZHS) was incorporated in the NR containing CP and Sb_2_O_3_, the UL-94 V0 was achieved. Menon et al. [28] found that the NR that was modified with a phosphorylated cashew nut shell liquid (PCNSL) prepolymer had good mechanical properties because the PCNSL played an important role as a softening agent. On the basis of PCNSL, they further prepared a bromo derivative of PCNSL (BrPCNSL), the flame retardancy of NR was improved significantly when filled with the BrPCNSL. In addition, the ATH as a typical inorganic flame retardant may significantly reduce the smoke density of NR, and play an efficient smoke-suppressing role [29]. Ismawi et al. [30] found that the LOI was improved to 22.6% when a small amount of antimony trioxide (7.5 phr) and decabromodiphenyl oxide (DBBO) was added in NR, and a UL-94 V1 rating was achieved.

Although halogen-containing flame retardants may play a flame-retarding role in NR, they would produce toxic substances and pollute the environment during the practical application. More seriously, some of these flame retardants may deposit in the organism and then bring about a terrible influence on the lifeform. Therefore, more and more attention was paid to new environmentally friendly halogen-free flame retardants.

#### 5.2.2. Phosphorus-Containing Flame Retardants 

Phosphorus-based flame retardants are regarded as a type of effective and environmentally friendly halogen-free flame retardant. Most of the current phosphorus-based additive flame retardants may act simultaneously in condensed and gaseous phases. In the condensed phase, phosphorous-based additive flame retardants may make the amount of carbonaceous residue or char increase during burning and then reduce the volatized decomposition products. In the gaseous phase, some phosphorus-based additive flame retardants may produce a lot of free radicals during the thermal decomposition process, and they may react with the free radicals which are generated from polymers, then the free-radicals-supported combustion of polymers might be stopped due to the lack of fuel [31,32]. In Menon et al.’s report [33], different amounts of 2-ethyl hexyl diphenyl phosphate was added in NR by a two-roll mixing mill; the LOI of NR composite increased from 17.0% to 17.5% at 15 phr of the additive. 

In fact, the phosphorus-based flame retardants may not be sufficient to improve the flame retardancy of NR when they are used alone. The reason is that the chemical composition of NR does not contain oxygen element and it is very difficult to char during burning [16]. In the case of there being not enough condensed and gaseous flame-retarding action for phosphorus-based flame retardants, the flame retardancy of NR will be unsatisfactory. In addition, the compatibility between phosphorus-based additives and the NR matrix is poor, so the mechanical properties of flame-retarding NR are also not ideal. Therefore, phosphorus-based flame retardants need to be further developed to achieve high flame-retarding efficiency in NR. 

#### 5.2.3. Nitrogen-Containing Flame Retardants

**Figure 5:**
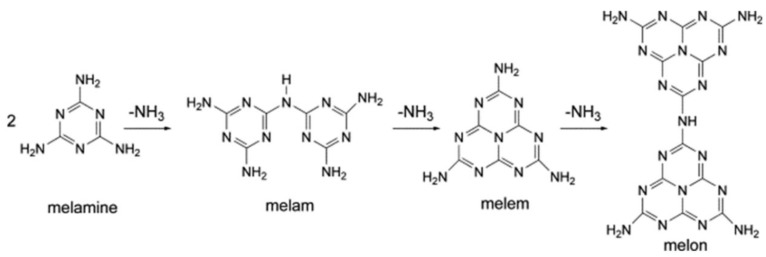
Thermal decomposition of melamine [16].

Melamine and its derivatives are the most typical nitrogen-containing flame retardants. For melamine, the nitrogen content is relatively high, up to about 67.0 wt.%. Under high temperatures, melamine will decompose and produce ammonia, melem, melam, melon, etc. (Figure 5) [34], and the formed gases may dilute the concentration of oxygen and combustible gases, endowing the polymer with flame retardancy. Besides, the condensation products of small molecules would form a protective layer in the condensed phase to resist further erosion of heat flow. Consequently, nitrogen-containing flame retardants may play a significant role not only in the gas phase but also in the condensed phase [35]. Melamine salts occupy a very important position in nitrogen-based flame retardants; its flame-retardant mechanism is different from that of melamine. During the thermal decomposition, melamine salts may produce melamine, and a small amount of melamine can be volatilized into the gas phase, but most of the formed melamine was deposited in the condensed phase. Therefore, the melamine salts play a flame-retarding role mainly depending on their condensed phase action. 

Currently, melamine and its salts play a dominant part in nitrogen-containing compounds [36]. Generally speaking, in terms of the flame-retarding efficiency, it is not high for melamine and its salts, so they are rarely used alone. The LOI of NR was increased by 47% when 17.0 wt.% of melamine cyanurate (MC) along with 1.0 wt.% antimony trioxide and 5.0 wt.% chlorinated wax was added [37]. 

#### 5.2.4. Phosphorus/Nitrogen Flame Retardants

In current research, phosphorus/nitrogen (P/N) flame retardants are considered to be one of the most important systems to achieve highly efficient halogen-free flame retardance of polymers [38,39]. Among the P/N system, intumescent flame retardants (IFR) have been paid much attention in the past ten years due to their high flame-retarding efficiency [40]. Upon heating, IFR interacts with the substrate and form foamed cellular charred layers at the surface, which may protect the underlying material from the erosion of heat flux or fire. Moreover, the transfer of oxygen and combustible gases will be blocked by the formed intumescent char layer [41,42,43].

**Figure 6:**
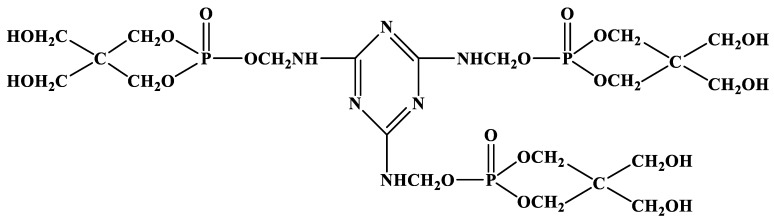
The structure of intumescent flame retardant (IFR) that was synthesized by Wang et al. [44].

In general, the IFR system is commonly composed of three components, which are an acid source, a carbonizing agent, and a blowing agent. The acid source is an inorganic acid or a compound capable of generating an acid upon heating to 100–250 °C, such as ammonium polyphosphate (APP); the carbonizing agent is a type of carbon-rich component that may be dehydrated via the catalytic action of the formed acid, and accompanied by cross-linking and graphitizing, such as pentaerythritol (PER); the blowing agent may release volatile products or gas above a certain temperature, and the most typical representative is melamine (MA). The acid must be released at a temperature below the decomposing temperature of the carbonizing agent, and the dehydration should be carried out near the decomposing temperature of the polymer [16]. Similar to most of polyolefin, it is very difficult for NR to char during burning, so it is necessary to make full use of the carbonizing agent to generate the char. Wang et al. [44] synthesized a novel IFR (Figure 6) containing phosphorus and nitrogen and added it into NR. The influences of the prepared IFR on flame retardancy, mechanical properties, and curing characteristics of NR were studied. When the content of IFR was 30 phr in NR, both an LOI of 21.5% and a UL-94 V1 rating were achieved. By increasing the content of IFR to 60 phr, there was a little improvement in the LOI, but the UL-94 V0 rating was achieved. In addition, they synthesized a kind of hyperbranched intumescent flame-retardant (HIFR) agent [45]. Compared with the IFR system, NR/HIFR showed better flame retardancy, higher tensile strength, and better wear-resistant properties under an equal loading of flame retardant. 

To enhance the efficiency of phosphorus/nitrogen flame retardants in flame-retarding NR, the microencapsulation method was developed in the past study, which may not only promote the flame-retarding efficiency, but also improve the compatibility between the IFR and NR substrate and further promote the mechanical properties of flame-retarding NR. For the microencapsulation method, both single-layered microencapsulation [46,47] and double-layered microencapsulation [48,49,50] were developed. For example, Wang et al. [47] synthesized a kind of microencapsulated intumescent flame retardant (MIFR) agent and found that the MIFR/NR achieved a UL 94 V-0 rating, but IFR/NR only achieved a UL 94 V-1 rating in the case of equal amount of flame retardant. Besides, the mechanical properties of IFR/NR were also improved after the microencapsulation of IFR. According to the result presented above, it can be concluded that the microencapsulation method is effective to enhance the flame retardancy of phosphorus/nitrogen flame retardants and reduce their damage to mechanical properties of NR. 

**Figure 7:**
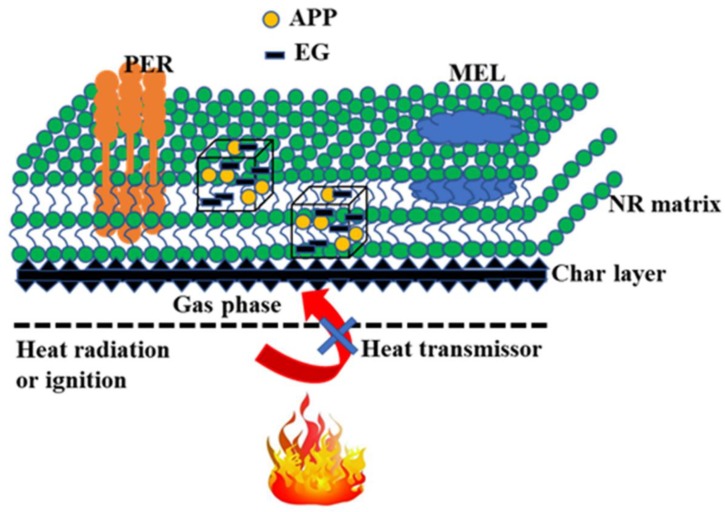
Schematic of flame-retardant mechanism for NR/IFR/expanded graphite (EG) [50].

In order to further improve the flame-retarding efficiency of IFR, synergistic agents are always added in IFR, and the number of synergistic agents is very low. For example, Liu et al. studied the effect of 4A zeolite on different properties of IFR-filled NR systems [51], and the result demonstrated that 0.3 phr of 4A zeolite make the cross-linking density, elongation at break, wear resistance, and LOI of NR increase, indicating that 4A zeolite is a kind of effective synergistic agent. Moreover, other synergistic agents have also been reported in NR/IFR systems, such as expanded graphite (EG) [50], graphene oxide (GO) [52], etc. In the case of adding these synergistic agents, the UL-94 rating of NR could be improved to V-0 and the LOI also increased significantly. Taking EG, for example, the flame-retardant mechanism is shown in Figure 7. EG could prevent the heat transmission to the inside of the substrate effectively by generating a thick char layer due to the thermal expansion effect, and the expanded char layer played an important role during the burning process of NR. 

#### 5.2.5. Inorganic Flame Retardants 

Inorganic flame retardants have been widely used due to their unique advantages, in which metal hydroxides are the most commonly used flame retardants, mainly including ATH and MH. The flame-retardant mechanism consists of two parts: in the condensed phase, flame retardants decompose and absorb a lot of heat, reducing the internal temperature of the substrate to slow down its pyrolysis rate, and the pyrolysis products would shield the surface of the substrate, which may act as a barrier to resist heat and oxygen. In the gas phase, the released water can absorb heat and also dilute the combustible gas and oxygen during the decomposition of flame retardants [53,54,55]. Both actions presented above may efficiently achieve the flame retardancy of polymers. Both ATH and MH are competitive flame retardants because of their low price and non-toxicity. ATH is usually used in halogen-free flame-retardant insulating or cable sheathing materials, showing good performance especially in suppressing smoke [56,57,58]. The decomposing temperature of ATH is about 180–200 °C (Equation (2)), so ATH is limited in polymers with low processing temperatures, such as ethylene-vinyl acetate (EVA) [59,60,61], ethylene propylene diene rubber (EPDM) [62], polypropylene (PP) [63,64], and linear low-density polyethylene (LDPE) [58], etc. As mentioned before, the processing temperature of NR is lower than the endothermic decomposing temperature of ATH, so ATH is a kind of commonly used flame retardant for NR. However, the flame retardant efficiency of ATH is low, so a high content (40–70 wt.%) must be added to meet the requirement of different products in flame retardancy [65]. Moreover, the mechanical properties of flame-retarding NR with a high content of ATH are easily deteriorated dramatically. Of course, this problem can be solved to some extent by surface modification. In addition, a small amount of ZHS may create an obvious synergistic effect with ATH to improve the fire resistive performance of NR [66]. The research result showed that 2 phr of ZHS made the LOI of NR containing 50 phr of ATH increase to 30.7% from 28.9% and the smoke density was decreased by 33.0%. Myers et al. [67] studied the effect of ammonium pentaborate, zinc borate, aluminum hydroxide, and calcium carbonate on the LOI of carbon black filled natural rubber products. The result shows that all the additives had a slight effect on the flammability as measured by LOI, and 25 phr of zinc borate or calcium carbonate can make the LOI of NR increase from 17.5% to 18.0%. In addition, 25 phr of ATH or ammonium pentaborate can promote the LOI of NR to 19.0%. ATH was also used to enhance the flame-resistant properties of thermoplastic vulcanizates (TPVs), such as NR/PP [68].

(2) $$2\mathrm{Al}{\left(\mathrm{OH}\right)}_{3}\;\left(s\right)\;\frac{\underline{180-200{}^{\circ}C}}{\;}{\mathrm{Al}}_{2}O_{3}\;\left(s\right)+3H_{2}O\;g$$

The initial decomposing temperatures of MH and ATH are remarkably different. For MH, its endothermic decomposition occurs between 300 and 320 °C, so it may be used in polymers with a higher processing temperature compared with ATH. It is known that the peak value of decomposing temperature for NR is about 380 °C. Once the MH is added in NR, all the water resulting from the thermal decomposition of MH will release before 380 °C, which may absorb the produced heat of the NR system during thermal decomposition, showing a potential value in fabricating flame-retarding NR. However, there is no responding report concerning the application of MH in NR alone. Chen et al. [69] studied the flame retardancy of hydrogenated nitrile butadiene rubber (HNBR)/ethylene-vinyl acetate rubber (EVA)/magnesium hydroxide (MH) system, and found that changing the ratio of two kinds of rubbers would have an influence on the LOI of the HNBR/EVA/MH system, but all samples achieved a UL-94 V-0 rating by adding 150 phr MH, illustrating that MH may play an important role in flame-retarding rubber.

(3) $$\mathrm{Mg}{\left(\mathrm{OH}\right)}_{2}\;\left(s\right)\;\frac{\underline{300-320{}^{\circ}C}}{\;}\mathrm{MgO}\;\left(s\right)+H_{2}O\;\left(g\right)$$

#### 5.2.6. Nano Flame Retardants

Nanoparticles acting as a class of flame-retardant additives have showed their unique advantages, and the synergistic flame retardation via nanoparticles has been a hotspot in recent years. A large number of studies indicate that polymer nanocomposites should be the most promising flame-retardant material in the future [70,71,72]. Generally speaking, a low content of nano flame retardants (normally less than 5.0 wt.%) may have a significant effect on the flame retardancy of polymers, especially MLR, PHRR, and HRR, which have a significant reduction in the presence of a small amount of nano flame retardants. Moreover, nano flame retardants are superior to almost all current flame retardants in terms of improving the physical properties of polymer substrates. Since montmorillonite (MMT) nanoparticles were used to fabricate a flame-retarding PA6 nanocomposite by Toyota Corporation (Japan) in 1976, more and more nano flame retardants have been introduced to prepare high-performance flame-retarding polymers [73], such as graphite oxide (GO) [74,75], layered doubled hydroxides (LDH) [76], carbon nanotubes (CNTs) [77], polyhedral oligosilsesquioxane (POSS) [78], fullerene (C60) [79], etc. The flame-retardant mechanisms of nano flame retardants are very complicated, in which the interaction between nano flame retardant and substrate is the critical factor, thus, the flame-retardant mechanism for different nano flame retardants in different polymers may be different due to various structural features of the polymer matrix and nanoparticles. In fabricating high-performance flame-retarding NR composites, the most common nanomaterials include MMT, carbon nanotube, graphene, HNT, etc. In the following part, their application in NR will be introduced briefly.

**Figure 8:**
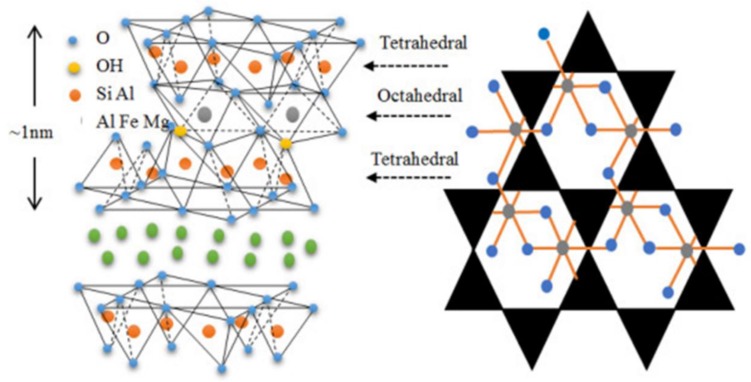
Schematic representation of the montmorillonite (MMT) structure [85].

Montmorillonite (MMT) is a kind of phyllosilicate mineral and it is characterized by a nanolayered structure, originating from the structural family of 2:1 clay layer. Its crystal structure (ca. 1 nm in thickness) consists of stacked layers, and each layer is composed of two O–Si–O tetrahedral sheets sandwiching one O–Al·(Mg)–O octahedral sheet (ca. 100 × 100 nm in width and length). As shown in Figure 8, these layers assemble themselves together by stacking up in a recurrent manner and form a flipping-book-like structure with a regular gap within consecutive layers, and finally, MMT retains a platelet structure [80]. Generally, a small amount of MMT may remarkably enhance the mechanical properties [81], thermal stability [82], barrier properties [83], flame retardancy [84], etc., for NR. Uniform dispersion of MMT in NR is a crucial prerequisite for ensuring excellent properties of the fabricated composite. Many researchers did a lot of work related to modifying the MMT to promote its dispersity, such as grafting with a silane coupling agent, coating, etc. Accordingly, the extent of exfoliation, content, and distribution of MMT nanoparticles in NR have a significant impact on the properties of nanocomposites.

**Figure 9:**
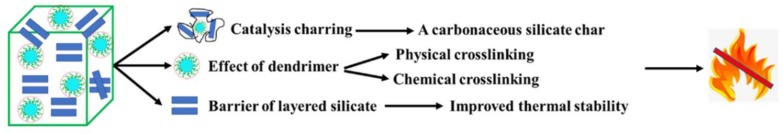
Flame-retardant mechanism of NR/FR-DOMt nanocomposite [81].

Khanlari et al. [86] prepared organic montmorillonite/NR intercalated nanocomposites by melt blending, and the result showed that 3.0 wt.% of OMMT improved the thermal stability and flame retardancy of NR, the onset decomposition temperature was promoted to 290.5 from 252.5 °C, and both HRR and PHRR had an obvious reduction compared with the corresponding values of pristine NR. Wang et al. [82] did a lot of work on MMT in preparing flame-retarding NR, in which most of the work focused on the modification of MMT to increase the interlayer spacing and enhance the intercalation and exfoliation of MMT in polymers. For instance, Wang et al. [84] modified MMT using a kind of hyper-branched polymer via the condensation polymerization and then prepared the NR nanocomposites by mechanical mixing. In studying the effect of modified MMT on the fire performance and mechanical properties of NR, they found that both tensile strength and the elongation at break were increased by 47.0% and 40.0% at 20 phr of modified MMT, and the LOI of NR was also increased to some extent. The reason for the enhancement in mechanical properties and fire resistance is that hyper-branched polymers caused the “anchor” effect to decrease the number and size of voids in the NR matrix. In another work, Wang et al. prepared dendrimer modified flame-retardant organic montmorillonite (FR-DOMt) [81]. For the prepared NR composite containing FR-DOMt, it showed excellent fire resistive performance in cone calorimeter and horizontal burning (HB) tests. The detailed flame-retardant mechanism of NR/FR-DOMt is shown in Figure 9. In synergistic flame retardation using the MMT, Wang et al. prepared a novel flame retardant through the combination of tricresyl phosphate (TCP) and OMMT, the LOI of the NR containing 10 wt.% of TCP and 10 wt.% of OMMT was 34.0%, which is much higher than that of pure NR (16.0%); furthermore, the mechanical properties, thermal stability, and wear resistance of the NR/TCP/OMMT were improved compared with the corresponding values of NR containing only TCP or OMMT. In addition, they also studied the triaryl phosphate (TAP)/OMMT flame retardant system in NR, and the result showed that the fire retardancy, tensile strength, and elongation at break of NR were enhanced by the TAP/OMMT. The analysis result showed that TAP improved the compatibility of the NR/OMMT system and then led to the improvement of the overall performance of the composite. 

A carbon nanotube (CNT) was also used to improve the flame retardancy of NR in past research. CNT is a kind of hollow nanotube that is made of single or multiple rolled-up graphene sheets [87,88], and it owns an extremely high surface-to-volume ratio and aspect, and also possesses excellent mechanical, thermal, optical, electrical properties, etc., due to its unique structure [89,90]. In the flame-retardant field, the application of CNT has been developed. Except for the barrier effect, CNT may capture free radicals and make many polymers produce a continuous structured network during the burning process [91,92,93,94]. Cho et al. [95] prepared the NR composites filled with CNT and OMMT by melt mixing. Under an equal 5.3 wt.% of OMMT and CNT, the PHRR values of NR nanocomposites were reduced to some extent. Moreover, a slight synergistic flame-retarding effect was formed between OMMT and CNT during burning. 

Graphene is another kind of important carbon material. Due to its 2D hexagonal lattice structure and high specific surface area, graphene has many unique properties including high electrical conductivity, excellent mechanical strength, superb thermal conductivity, etc. [96,97]. Graphene has shown great potential in the flame-retardant field, and more and more polymer/graphene nanocomposites were prepared and studied deeply [98,99,100]. Graphene may act as a physical barrier to resist the escape of the pyrolysis products and prevent heat and oxygen transferring between interfaces [101,102]. Wang et al. [52] developed a novel flame-retardant synergistic agent through grafting MCM-41 onto graphene oxide (GO), named as GO-NH-MCM-41. As an assistant component to IFR, the flame-retardant effect is very impressive. In the presence of a small amount of GO-NH-MCM-41. The LOI value of NR/IFR reached 26.3% and the UL-94 rating was improved to a UL-94 V-0 rating. The analysis demonstrated that the improvement in flame retardancy should be attributed to the synergistic effect between graphene oxide and MCM-41. 

**Figure 10:**
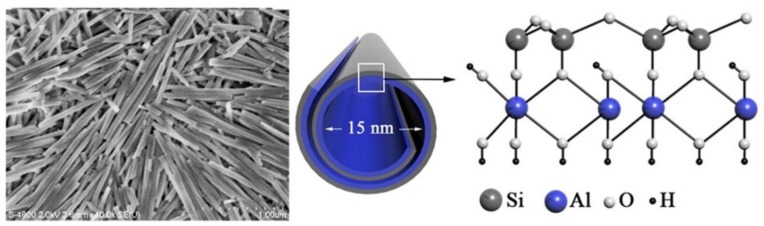
Schematic illustration of crystalline structure of halloysite nanotube (HNT) [114].

HNT became a popular nanomaterial due to its high surface area, aspect ratio, porosity, as well as high thermal stability. As shown in Figure 10, different from MMT, HNT is a kind of one-dimensional natural mineral clay which is a 1:1 aluminosilicate, and the chemical formula is Al_2_Si_2_O_5_(OH)_4_·2H_2_O. It exhibits a tubular or scroll-shaped structure that consists of one tetrahedrally coordinated Si (i.e., SiO_4_) and a sheet of octahedrally coordinated Al (i.e., AlO_6_) [103,104]. The interfacial interaction between HNTs and polymers can be tailored using chemical or physical approaches. Almost all polymers can be mechanically reinforced by HNTs, even at a low content (normally less than 5.0 wt.%) [105]. HNT may improve the flame retardancy of polymers mainly through physical action such as a barrier against the transport of heat and oxygen [106,107]. In the past work, HNT was widely used as a kind of effective flame retardant in different polymers such as poly (butylene succinate) (PBS) [108], PP [109], polyurethane [110], polyamide 6 [111], epoxy [112], etc. Tan et al. [113] modified the HNT with octadecylamine (ODA) and prepared the modified halloysite nanotubes (OHNT)/epoxidized natural rubber with about a 50.0% epoxidation level (ENR-50) of nanocomposites via the solvent casting technique, the physicochemical properties and flame retardancy of the composites were studied. In burning tests, the prepared OHNT/ENR-50 nanocomposites achieved self-extinguishing, and the LOI values were 72.0% and 48.0% for the corresponding OHNT5/ENR-50 and OHNT1/ENR-50, reaching about 1.4–2 times higher than the vulcanized NR with 0.5 phr of zeolite and 80 phr of APP/PER/melamine. Therefore, HNT as a naturally available flame retardant has a great potential value in NR.

Compared with traditional flame-retardant systems, the nano flame-retardant system has its unique advantages such as low addition, high efficiency in some aspects, as well as low damage to mechanical properties of the polymer matrix, etc. However, it is difficult for NR to reach a UL-94 V-0 rating in the vertical burning test in the presence of nanoparticles alone, and the flame-retarding efficiency and mechanical effect of nanoparticles on NR are easily affected by the distribution and dispersion. Therefore, much work is still to be carried out in future work. 

## 6. Concluding Remarks and Outlook

According to the review presented above, it is known that intrinsic flame-retardant NR owns its unique advantages in endowing the substrate with permanently flame retardancy and mechanical properties. However, the preparation process is very complicated and the variety of effective flame-retardant monomers is very limited. Halogen-containing NR shows excellent flame retardancy via radical trapping action during burning. However, the toxic and corrosive hydrogen halide gas that is produced during burning poses a great threat to the safety of people. For the halogen-free flame retardants with higher safety in health and environmental pollution than halogen-containing flame retardants, it dominates the current research direction on flame retardant, in which phosphorus/nitrogen synergistic flame retardants have high flame-retarding efficiency, but always remarkably deteriorate the mechanical properties of polymer matrix due to their poor compatibility with NR, and many researchers have developed various methods to optimize such system, including microencapsulation, etc. Until now, phosphorus/nitrogen synergistic flame retardants should be the most promising candidates for the current popular inorganic flame retardants MH and ATH. How to further improve the flame-retarding efficiency, water resistance, and compatibility is still a big challenge for fabricating high-performance flame-retarding polymers. Therefore, some work still needs to be carried out in future work. For the current popular inorganic endothermically decomposing fillers, they have remarkable advantages such as low toxicity, low smoke, abundant source, and low price, so they are likely to remain the mainstay of halogen-free systems for some time. However, the mechanical loss and processing difficulty caused by the high content (40–70 wt.%) of inorganic flame retardants are still not solved. Nanocomposites have special advantages such as low addition, high efficiency in promoting the LOI, slight damage to mechanical properties of the polymer matrix, etc., but the weak effect on burning under the low-energy heat source and uncontrollable dispersion of nanomaterials are unsolved problems. 

Although much progress was made for fabricating flame-retarding NR, no perfect system was obtained currently. Different flame-retarding systems show their unique advantages and disadvantages. In future work, the emphasis on each category of a flame-retarding system is to develop its advantage and simultaneously overcome its disadvantage to prepare flame-retarding NRs with excellent overall performance. 

## Figures and Tables

**Table 1:** Classification for the rating in the UL 94 burning test (according to an international standard IEC 60695-11-10).

Evaluation Standards	Fire Classification		
	V-0	V-1	V-2
Number of samples	5	5	5
Burning time (s)	2	2	2
The flame burning time (s) of each sample after leaving fire is no more than	10	30	30
The flame burning time (s) of each group of 5 samples during 10 times of ignition after leaving fire is no more than	50	250	250
The flameless burning time (s) of each sample after the second ignition and leaving fire is no more than	30	60	60
The phenomenon that each sample has flame or flameless combustion spread to the fixture	NO	NO	NO
Each sample dripping ignited cotton wool	NO	NO	Yes

**Table 2:** Typical endotherm hydrated compound via decomposing [19,20].

Name	UltraCarb	Hydroe Magnesite	Magnesium Hydroxide	Huntite	Aluminum Hydroxide
Chemical formula	hydromagnesite: huntite (approx. 60:40)	Mg_5_(CO_3_)_4_ (OH)_2_·4H_2_O	Mg(OH)_2_	Mg_3_Ca(CO_3_)_4_	Al(OH)_3_
H ^b^ (J·g^−1^)	990	1300	1450	980	1300
T_onset_ (°C)	220–240	220–240	300–320	~400	18,200
Relative contribution fire retardant effects (%)					
Filler	14	10	19	20	9
Endotherm	57	56	56	58	55
Gas	18	21	15	13	23
Residue	12	14	9	9	13

^b^ the “H” means the enthalpy of decomposition.
